# Supplementary material for: Molecular and Spatial Organization of the Primary Olfactory System and its Responses to Social Odors
Source: bioRxiv. 2025 May 2:2025.05.02.651832. Preprint. [Version 1] doi: 10.1101/2025.05.02.651832 (PMC12248032; doi:10.1101/2025.05.02.651832)
Supplement: Supplement 1 [file NIHPP2025.05.02.651832v1-supplement-1.pdf]

bioRxiv preprint doi: https://doi.org/10.1101/2025.05.02.651832; this version posted May 2, 2025. The copyright holder for this preprint (which was not certified by peer review) is the author/funder, who has granted bioRxiv a license to display the preprint in perpetuity. It is made available under aCC-BY 4.0 International license.

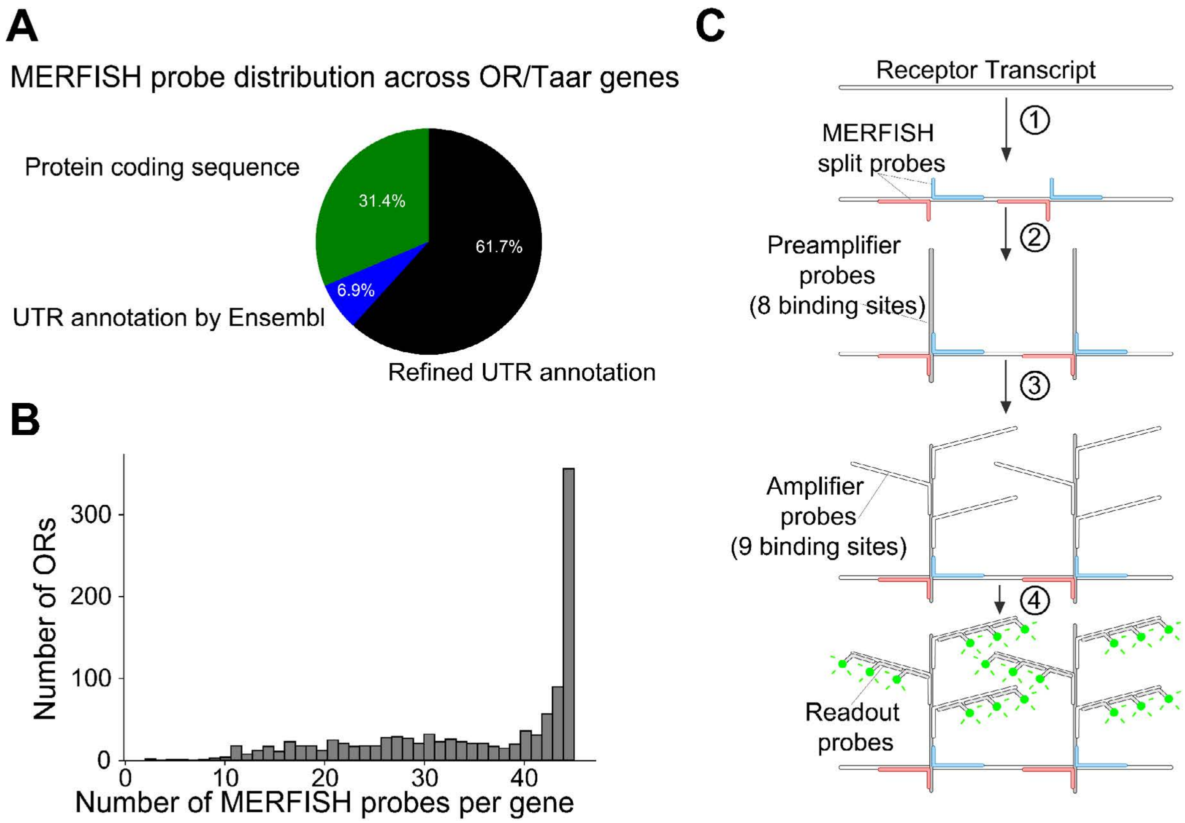

Figure S1. Modification to the MERFISH probe design to enable genome-scale OR/Taar targeting. (A) Pie chart with the percentage of the MERFISH encoding probes targeting the receptor coding region (green), the Ensemble annotated UTR regions(blue) and the extended annotation (from [40]) of the receptor UTR (black). **(B)** Histogram with the distribution of the number of split-pairs of encoding probes across OR/Taar genes. **(C)** Schematic of the split-branched amplification scheme. In the 1st step pairs of encoding oligonucleotide probes are first hybridized to the receptor transcripts. In the 2nd step each pair of encoding probes colocalized onto the transcript allows the stable binding of a 200-nt single stranded DNA probe, called a preamplifier probe. The preamplifier contains eight 20-nt repetitive binding sequences. In the 3rd step, a similarly designed 200-nt single stranded DNA probe, called an amplifier probe, binds to the repetitive binding sites of the preamplifier. Finally in the 4th step, 30-nt fluorescent oligos called readout probes bind the repetitive sequences of the amplifier probes.

bioRxiv preprint doi: https://doi.org/10.1101/2025.05.02.651832; this version posted May 2, 2025. The copyright holder for this preprint (which was not certified by peer review) is the author/funder, who has granted bioRxiv a license to display the preprint in perpetuity. It is made available under aCC-BY 4.0 International license.

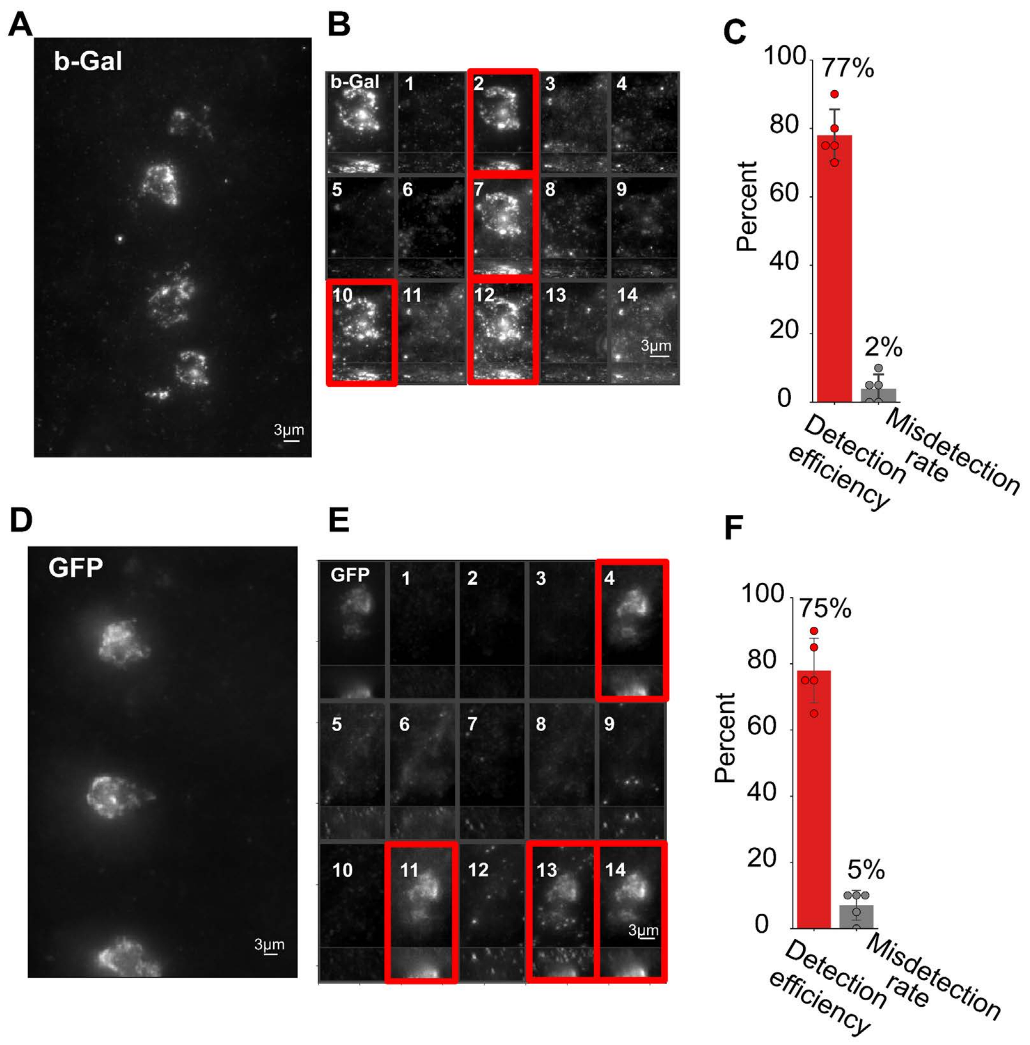

Figure S2. Quantifying the detection efficiency and accuracy of MERFISH using transgenic lines. (A) An image with the fluorescent signal of smFISH probes hybridized to β-Gal in a section of the MOE of a Olfr17-IRES-tau-lacZ transgenic animal. **(B)** Max-projection images of a β-Gal positive neuron showing the signal of the β-Gal probes (top left panel) and the MERFISH signal across 14 readout cycles. Readouts 2, 7, 10 and 12 (highlighted in red) correspond to the 4 readout probes binding to the MERFISH encoding probes designed for the Olfr17 transcript. **(C)** Bar plots marking the detection efficiency and misdetection rate for the Olfr17 transcript. The detection efficiency is defined as the fraction cells expressing β-Gal identified as Olfr17 positive via MERFISH. The misdetection rate is defined as the fraction of cells identified as Olfr17 positive via MERFISH which do not express β-gal. Quantification was performed across 2 MOE sections. The mean and standard deviation are plotted. **(D),(E)** and **(F)** Same as (A), (B) and (C), respectively, for Olfr16 in an Olfr16-IRES-tau-GFP transgenic animal.

bioRxiv preprint doi: https://doi.org/10.1101/2025.05.02.651832; this version posted May 2, 2025. The copyright holder for this preprint (which was not certified by peer review) is the author/funder, who has granted bioRxiv a license to display the preprint in perpetuity. It is made available under aCC-BY 4.0 International license.

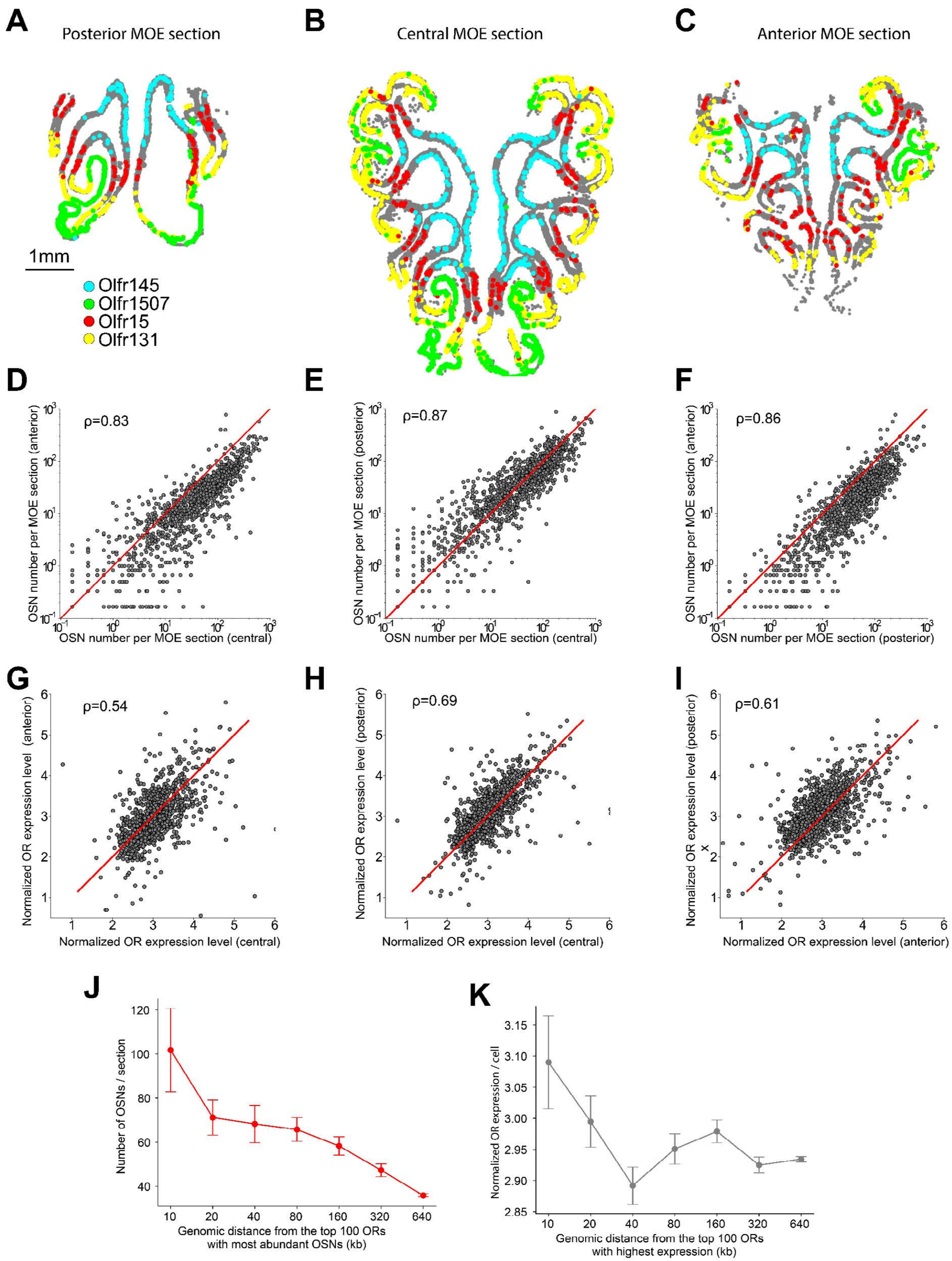

Figure S3. OSN abundance and OR expression level per cell are similar across the anterior-posterior axis of the MOE. (A), (B), (C) Representative coronal sections from the

bioRxiv preprint doi: https://doi.org/10.1101/2025.05.02.651832; this version posted May 2, 2025. The copyright holder for this preprint (which was not certified by peer review) is the author/funder, who has granted bioRxiv a license to display the preprint in perpetuity. It is made available under aCC-BY 4.0 International license.

posterior, central and anterior MOE, respectively, overlayed with OSN positions of 4 example ORs. **(D), (E), (F)** Correlations between the number of OSNs per section for each OR comparing anterior to central, posterior to central and anterior to posterior MOE sections respectively. **(G),(H), (I)** Same as (D), (E), (F) for the average OR expression for each OSN type. **(J)** Correlation between the average number of OSNs per section and the genomic distance from the top 100 ORs with most abundant OSNs. **(K)** Correlation between the average OR expression per cell and the genomic distance from the top 100 ORs with highest expression. Error bars mark the standard error of the mean.

bioRxiv preprint doi: https://doi.org/10.1101/2025.05.02.651832; this version posted May 2, 2025. The copyright holder for this preprint (which was not certified by peer review) is the author/funder, who has granted bioRxiv a license to display the preprint in perpetuity. It is made available under aCC-BY 4.0 International license.

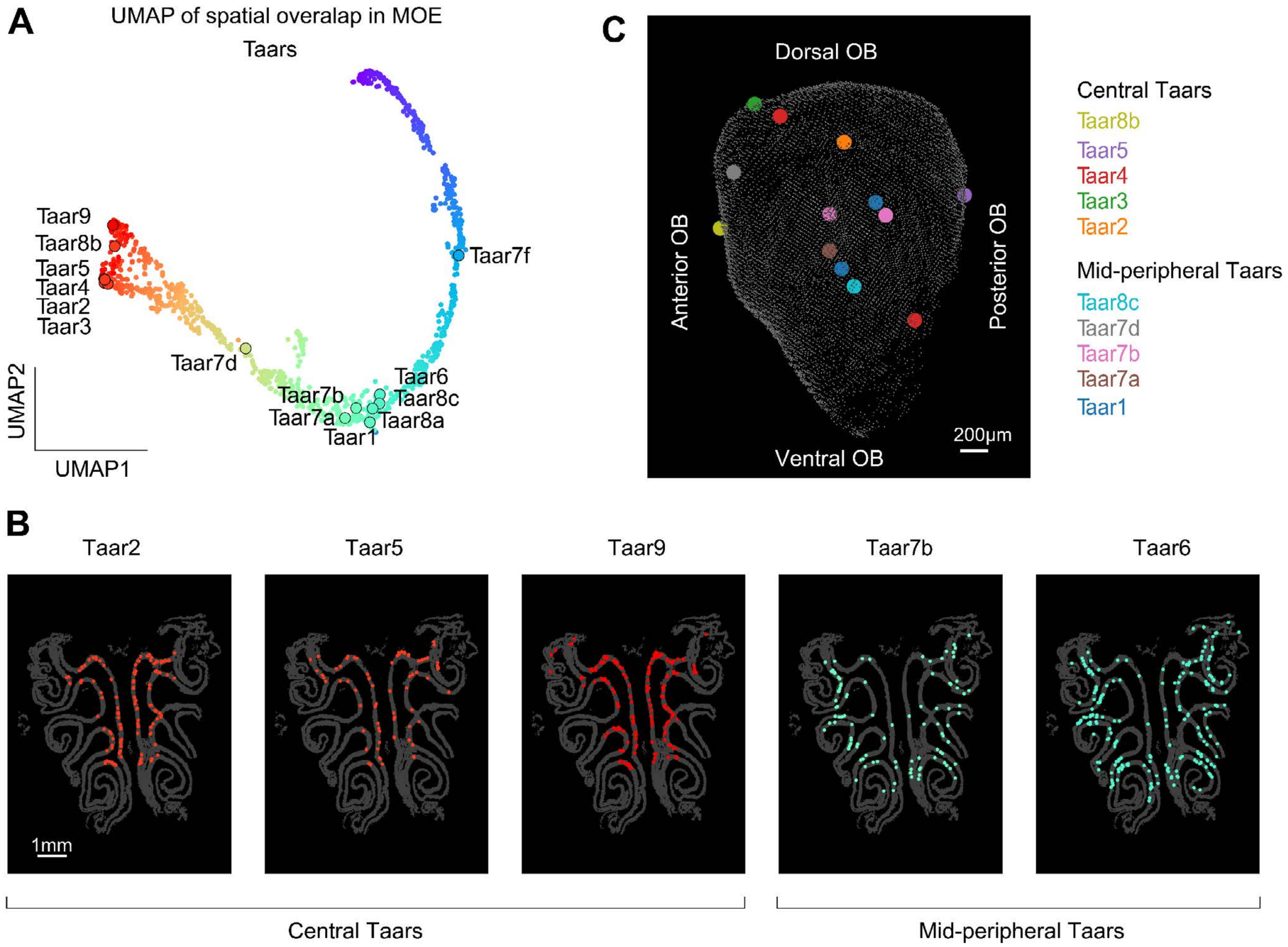

Figure S4. The spatial distribution of Taars within the MOE and OB. (A) UMAP representation of ORs based on their spatial overlap (reproduced from Figure 2B) which Taar genes overlayed. Taars are primarily located central or mid-peripheral MOE **(B)** Example images showing the spatial distribution of 3 Taar genes within the central MOE and 2 Taar genes within the mid-peripheral MOE. **C.** Image highlighting the Taar projections detected within the OB.

bioRxiv preprint doi: https://doi.org/10.1101/2025.05.02.651832; this version posted May 2, 2025. The copyright holder for this preprint (which was not certified by peer review) is the author/funder, who has granted bioRxiv a license to display the preprint in perpetuity. It is made available under aCC-BY 4.0 International license.

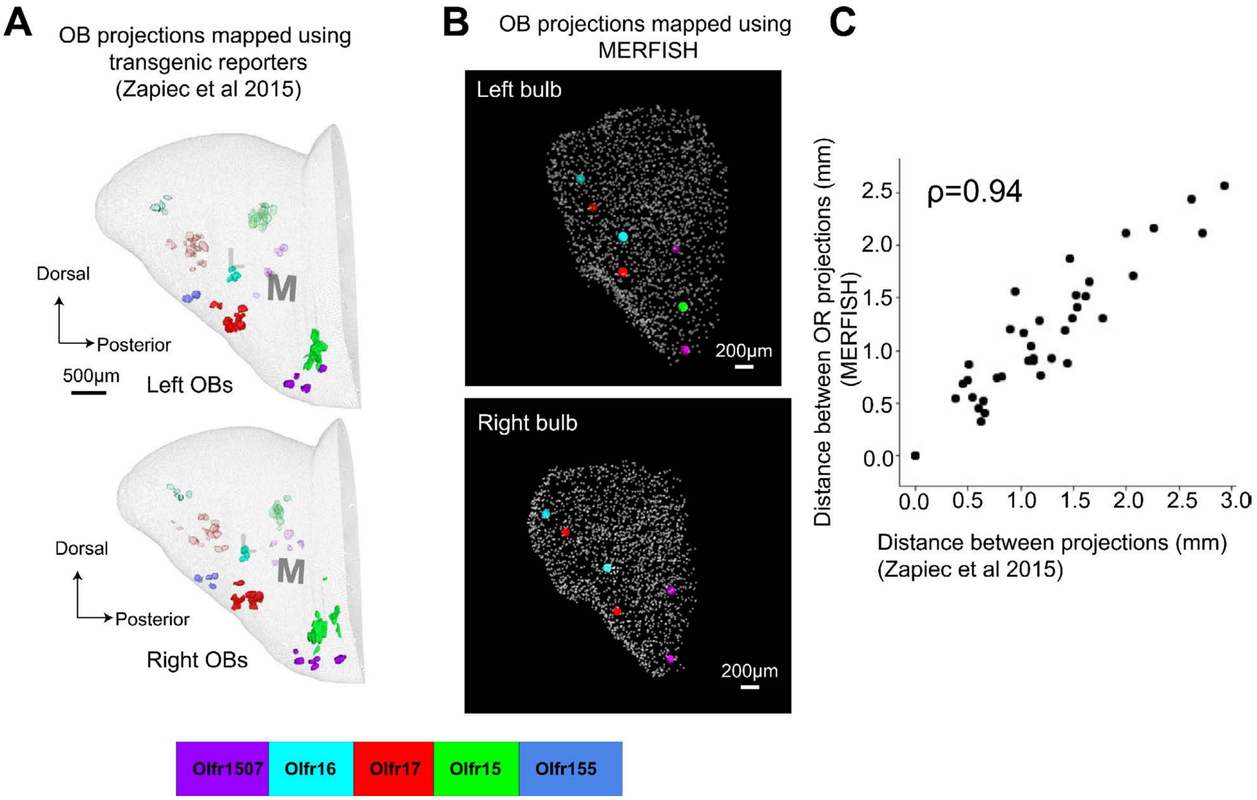

Figure S5. Comparing MERFISH identified OR projections with OR projections labeled in transgenic lines. (A) The 3D projections for 5 ORs identified by serial two-photon tomography in transgenic lines with fluorescently labeled ORs. The left and right bulbs of multiple animals were aligned to construct a unified map. This panel is reproduced from[20]. **(B)** MERFISH identified projections for the corresponding ORs across the left and right OB of a CD1 female mouse. **(C)** Correlation between the pairwise distances of OR projections identified in [20] and the corresponding pairwise distances of OR projections identified by MERFISH. Pearson correlation coefficient of 0.94.

bioRxiv preprint doi: https://doi.org/10.1101/2025.05.02.651832; this version posted May 2, 2025. The copyright holder for this preprint (which was not certified by peer review) is the author/funder, who has granted bioRxiv a license to display the preprint in perpetuity. It is made available under aCC-BY 4.0 International license.

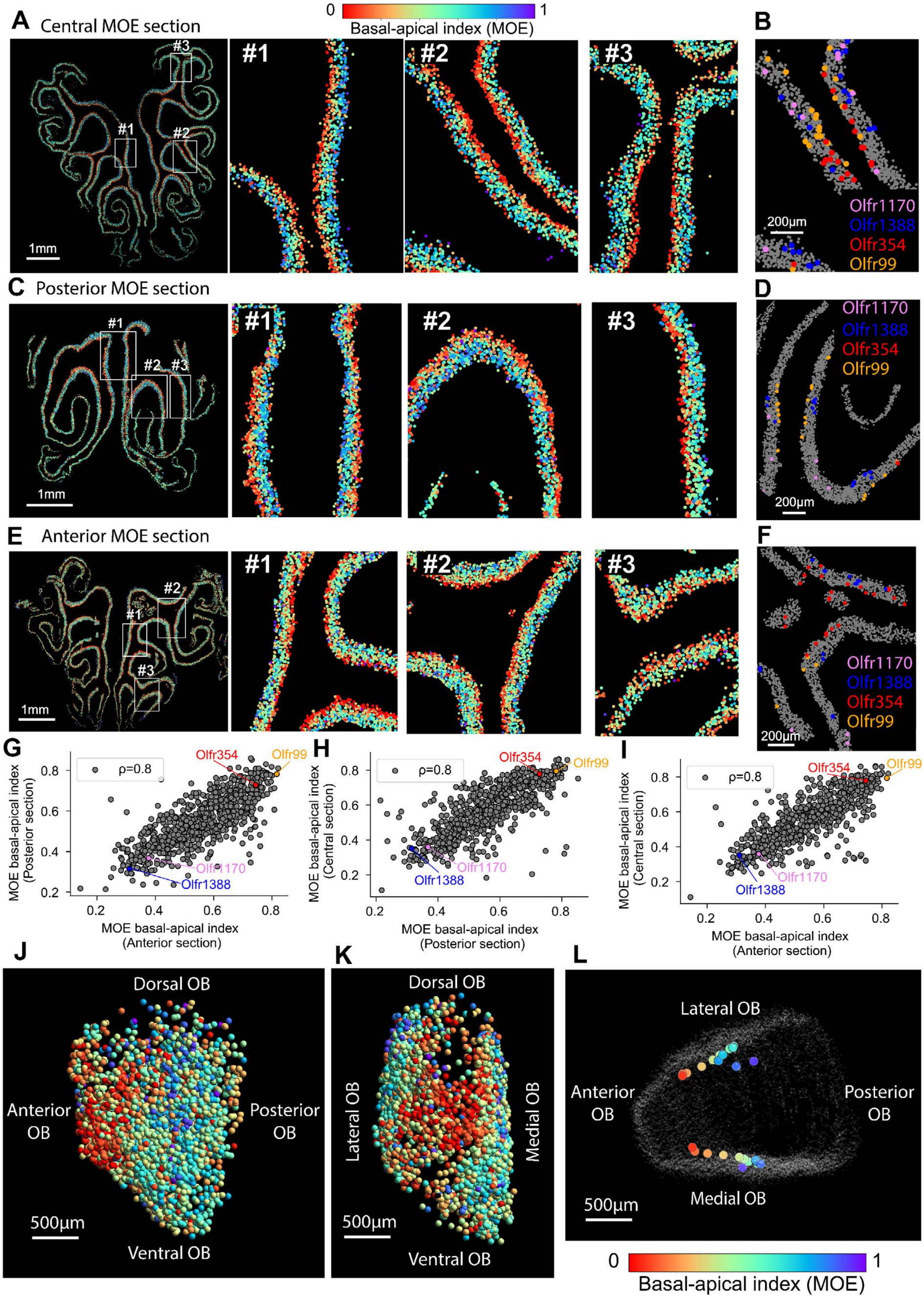

bioRxiv preprint doi: https://doi.org/10.1101/2025.05.02.651832; this version posted May 2, 2025. The copyright holder for this preprint (which was not certified by peer review) is the author/funder, who has granted bioRxiv a license to display the preprint in perpetuity. It is made available under aCC-BY 4.0 International license.

Figure S6. The basal-apical position of OSNs in the MOE correlates with the anterior-posterior position of their projections in the OB. (A) Example image of a central MOE section with OSNs labelled based on the average basal-apical position of each OR type. Zoom-ins are shown for 3 different MOE regions. **(B)** Zoom-in image on a MOE region with the positions of 4 example OSN types highlighted (Olfr354 and Olfr99 are more basal while Olfr1388 and Olfr1170 are more apical). **(C),(D)** and **(E),(F)** same as (A),(B) for a posterior section and an anterior MOE section respectively. **(G),(H),(I)** Pairwise correlation of the basal-apical position of OSNs between anterior, posterior and central sections of the MOE. **(J)** OB projections colored based on the basal-apical position of their corresponding OSNs in the MOE (medial side). **(K)** Same as (J) shown from a different view angle of the OB. **(L)** Image of the OB together with the average positions of OR projections binned based on the basal-apical positions of the corresponding OSNs in the MOE. Two parallel axes (one for medial projections and one for lateral projections) emerged along the anterior-posterior axis of the OB.

bioRxiv preprint doi: https://doi.org/10.1101/2025.05.02.651832; this version posted May 2, 2025. The copyright holder for this preprint (which was not certified by peer review) is the author/funder, who has granted bioRxiv a license to display the preprint in perpetuity. It is made available under aCC-BY 4.0 International license.

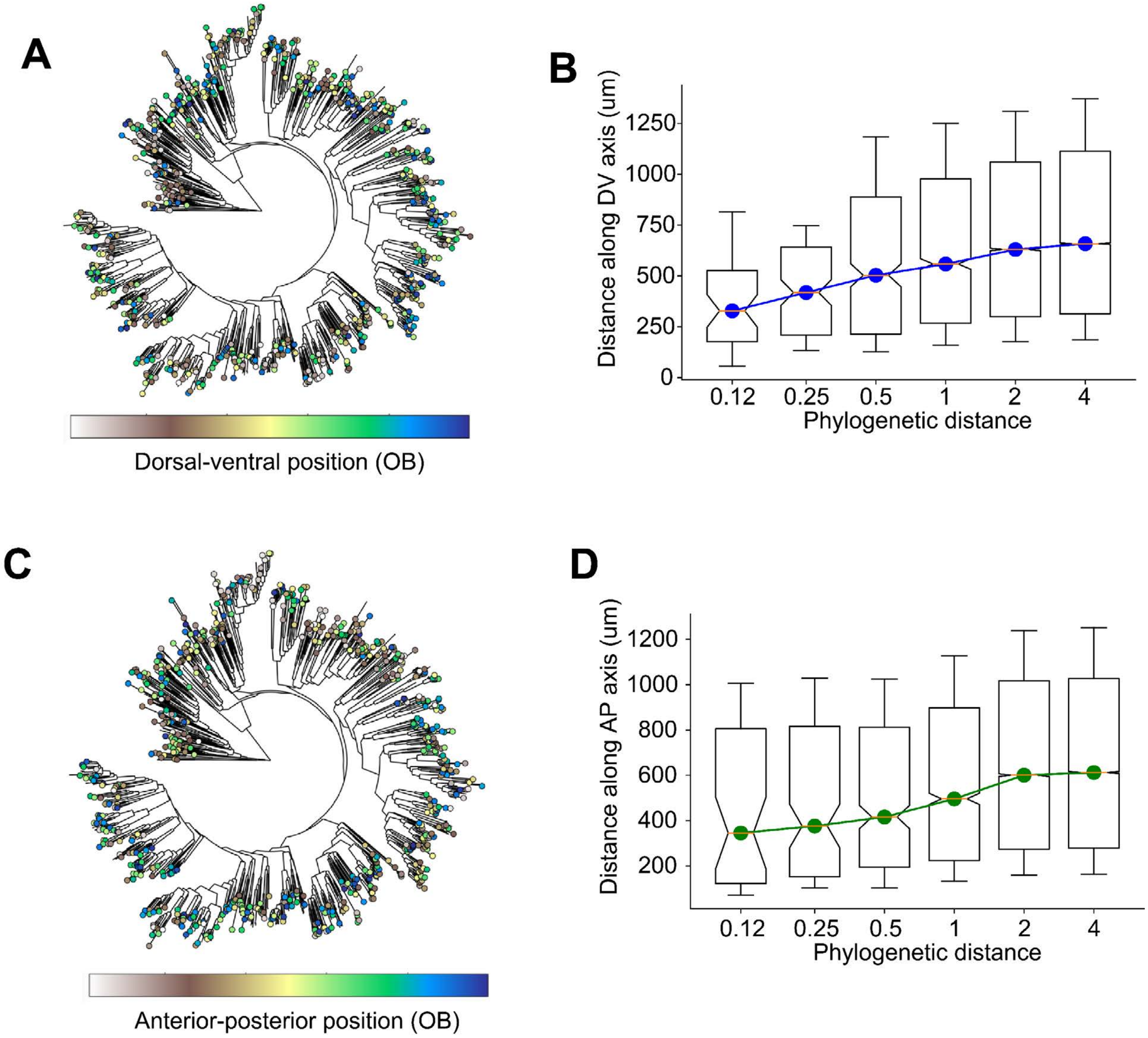

Figure S7. Relationship between OR phylogenetic distances and OB projections along dorsal-ventral and anterior posterior axes (A) Phylogenetic tree connecting *ORs* based on their sequence similarity, constructed as in[32]. Each node, representing an OR, is colored based on the dorsal-ventral position of the OB projection as indicated in the colormap. **(B)** Correlation between distances of *ORs* along the phylogenetic tree of pairs of ORs and distances along the dorsal-ventral projection axis. For each pair of ORs we calculated their distance along the tree in (A) and then binned these distances. For each bin we represent a box plot marking the median dorsal-ventral distances between the ORs within the corresponding phylogenetic distance range. Notches represent 95% confidence intervals, the boxes mark the first and third quartile and the whiskers mark 15th and 85th percentiles. **(C), (D)** Same as (A) and (B) respectively, for the anterior-posterior axis of the OB.

bioRxiv preprint doi: https://doi.org/10.1101/2025.05.02.651832; this version posted May 2, 2025. The copyright holder for this preprint (which was not certified by peer review) is the author/funder, who has granted bioRxiv a license to display the preprint in perpetuity. It is made available under aCC-BY 4.0 International license.

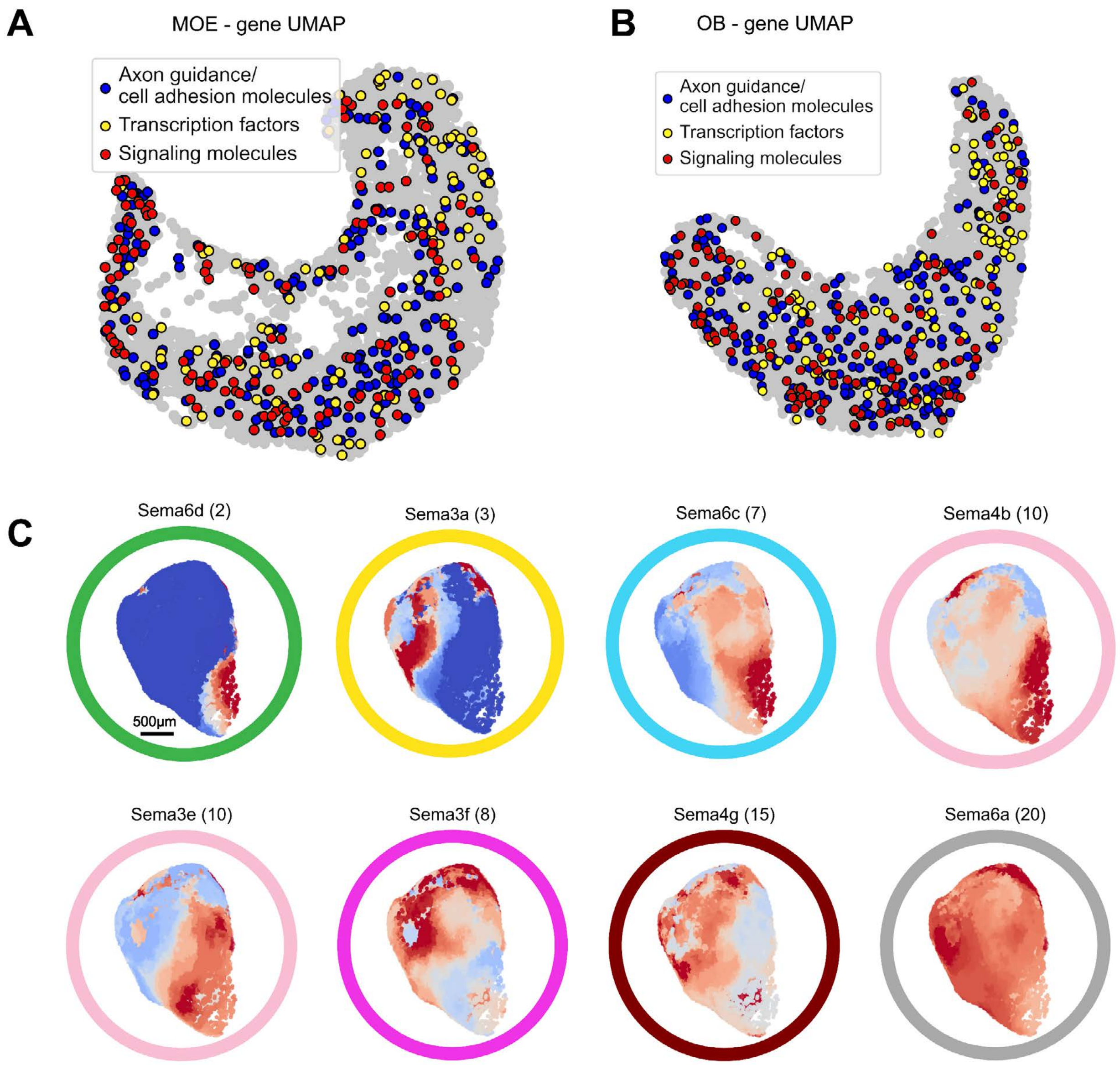

Figure S8. Variability of expression patterns imputed within MOE or OB for specific classes of genes . (A) UMAP representation of the MOE imputed spatial patterns (reproduced from Figure 4D) with the axonal guidance/cell adhesion molecules, transcription factors and signaling molecules overlayed. **(B)** Same as (A) for the OB UMAP reproduced from Figure 4F. **(C)** The imputed spatial patterns of the Semaphorin gene family in the OB.

bioRxiv preprint doi: https://doi.org/10.1101/2025.05.02.651832; this version posted May 2, 2025. The copyright holder for this preprint (which was not certified by peer review) is the author/funder, who has granted bioRxiv a license to display the preprint in perpetuity. It is made available under aCC-BY 4.0 International license.

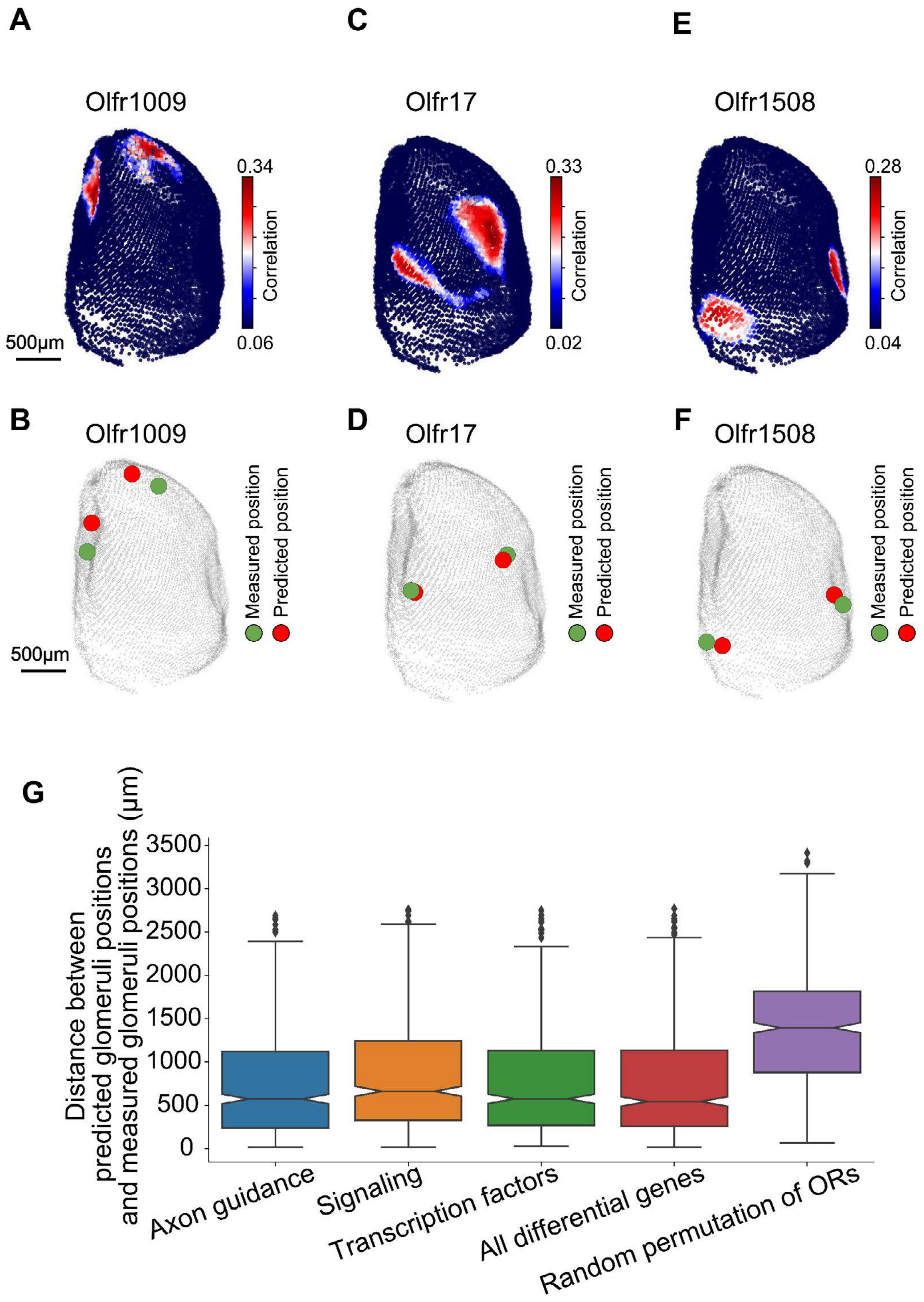

Figure S9. The predictability of OR projections in the OB based on the gene expression of OSNs in the MOE. (A) Correlation between the average gene expression of Olfr1009 expressing OSNs and the imputed expression across the OB from all the other ORs (excluding

bioRxiv preprint doi: https://doi.org/10.1101/2025.05.02.651832; this version posted May 2, 2025. The copyright holder for this preprint (which was not certified by peer review) is the author/funder, who has granted bioRxiv a license to display the preprint in perpetuity. It is made available under aCC-BY 4.0 International license.

Olfr1009). The correlation was computed across the top ~5000 differentially expressed genes for all points sampled on the glomerular layer. **(B)** OB image showing the projections of Olfr1009 measured by MERFISH (green) and the two points with the maximum medial and lateral correlations computed in (A) (red). **(C),(D)** Same as (A) and (B) for Olfr17. **(E), (F)** Same as (A) and (B) for Olfr1508. **(G)** Box plots showing the distances between the predicted glomeruli positions (red dots in (B), (D), (E)) and the MERFISH-identified glomeruli positions across the OR repertoire (green dots in (B), (D), (E)) when using different sets of genes for computing the correlation described in (A). Purple box plot shows the distances between the predicted glomeruli positions and the measured glomeruli positions using the correlation of the 5000 differentially expressed genes upon randomly shuffling the OR identities of the projections.

bioRxiv preprint doi: https://doi.org/10.1101/2025.05.02.651832; this version posted May 2, 2025. The copyright holder for this preprint (which was not certified by peer review) is the author/funder, who has granted bioRxiv a license to display the preprint in perpetuity. It is made available under aCC-BY 4.0 International license.

Supplemental Table Legends:

Table S1. Sequences of the MERFISH probes and associated primers/readouts used to target olfactory receptor, trace amine-associated receptor and immediate early genes.

Table S2. The median number of OSNs per section and the median normalized OR expression per OSN across female and male animals.

Table S3. The central-to-peripheral index in the MOE of the OSNs detected and their coordinate on the spatial overlap UMAP. The positions of all OSNs are also provided for 3 representative MOE sections from 3 animals.

Table S4. The 3D positions of all the glomeruli whose receptor identity was determined by MERFISH. Four olfactory bulbs were aligned in a single reference and the positions across them were concatenated. 3D positions defining the glomeruli layer surface of the reference olfactory bulb is also provided.

Table S5. The basal-to-apical index of each OSN type. Three MOE sections across different positions on the anterior-posterior axis were used to quantify the average basa-to-apical position of each OSN type. These positions were normalized such that 0 is the most basal position identified and 1 is the most apical.

Table S6. The response of each OSN type (defined as the fraction of OSNs of each type containing >5EGR1 transcripts per cell) for animals exposed to two concentrations of acetophenone.

Table S7. The response of each OSN type for animals exposed to different ethological cues.
